# Shear wave elastography in the evaluation of level VI lymph nodes in papillary thyroid carcinoma: combined with gray-scale ultrasound ex vivo

**DOI:** 10.1186/s12885-018-4897-1

**Published:** 2018-10-20

**Authors:** Wanying Chang, Lei Tang, Caiwei Lu, Min Wu, Man Chen

**Affiliations:** 1 Department of Diagnostic Ultrasound, Tong Ren Hospital, Shanghai Jiao Tong University School of Medicine, No. 1111 Xian Xia Road, Shanghai, 200336 China; 2 Department of Pathology, Rui Jin Hospital, Shanghai Jiao Tong University School of Medicine, No. 197 Rui Jin 2nd Road, Shanghai, 200025 China

**Keywords:** Shear wave elastography, Level VI lymph nodes, Papillary thyroid carcinoma, Gray-scale ultrasound

## Abstract

**Background:**

The evaluation of cervical lymph nodes is very important for patients with papillary thyroid carcinoma (PTC). Conventional ultrasound is recommended to assess the status of cervical lymph nodes but the diagnostic performance is not satisfying especially in level VI lymph nodes. Recently, shear wave elastography has shown great potential in diagnosis. Therefore, this study aimed at exploring the value of shear wave elastography in ultrasound evaluation for level VI lymph nodes in papillary thyroid carcinoma. Because Hashimoto’s thyroiditis may influence the diagnostic performance, a subgroup was also analysed that included only lymph nodes from PTC without Hashimoto’s thyroiditis.

**Methods:**

Eighty-Seven level VI lymph nodes from 22 consecutive patients with papillary thyroid carcinoma were evaluated by gray-scale ultrasound and SWE in condition of ex vivo before rapid frozen section. Gray-scale ultrasound and shear wave elastography indexes of metastatic and non-metastatic lymph nodes were evaluated by statistical analysis separately in all patients and in patients without Hashimoto’s thyroiditis. Indexes included long diameter, short diameter, short-to-long diameter ratio (S/L ratio), E_mean_, E_min_, E_max_ and E_SD_. The rapid frozen section result of each lymph node was used as gold standard to evaluate the diagnostic performance of gray-scale ultrasound and combination method which combined gray-scale ultrasound and SWE.

**Results:**

In all patients, significant indexes included short diameter (*p* = 0.009), S/L ratio (*p* = 0.003), E_max_ (*p* = 0.016) and E_SD_ (*p* = 0.006). In patients without Hashimoto’s thyroiditis, significant indexes included short diameter (*p* = 0.002), S/L ratio (p = 0.003), E_mean_ (*p* = 0.030), E_max_ (*p* < 0.001) and E_SD_ (*p* = 0.001). Combining gray-scale ultrasound with SWE, combination method had higher AUC than gray-scale ultrasound both in all patients (0.887 vs 0.841) and patients without Hashimoto’s thyroiditis (0.925 vs 0.866). Gray-scale ultrasound had higher AUC in patients without Hashimoto’s thyroiditis than in all patients (0.866 vs 0.841), which was the same with combination method (0.925 vs 0.887).

**Conclusion:**

Shear wave elastography can provide additional information for ultrasound evaluation of level VI lymph nodes in papillary thyroid carcinoma, especially in papillary thyroid carcinoma without Hashimoto’s thyroiditis.

## Background

The incidence of thyroid carcinoma is steadily increasing in recent years [1]. Papillary thyroid carcinoma (PTC) is the most common histological type and tends to metastasize to cervical lymph nodes [2]. Cervical lymph node metastasis of PTC is one of the important risk factor for tumor recurrence and generally occurs firstly in the level VI lymph nodes which are also called central cervical lymph nodes [3, 4]. Therefore, level VI lymph nodes were also considered as sentinel lymph nodes whose status can predict lateral involvement in PTC and impact the determination of tailoring treatment for patients [5]. Thus, the evaluation of level VI lymph nodes status is essential.

Preoperative Ultrasound for cervical lymph nodes, including central and lateral compartments lymph nodes, is strongly recommended by American Thyroid Association (ATA) to all patients undergoing thyroidectomy due to malignant or suspicious thyroid nodules [5]. However, the diagnostic performance of conventional ultrasound in cervical lymph nodes is not satisfying, especially in level VI lymph nodes [6, 7]. Other imaging techniques may provide additional information to help diagnosis.

Shear wave elastography (SWE) is a kind of ultrasound elastography which can quantitatively measure tissue elasticity and is a good complementary tool to B-mode ultrasound [8]. It has showed bright prospect in the evaluation of cervical lymph nodes due to its high reproducibility and high diagnostic performance (especially in group of sub-centimeter cervical lymph nodes) [9]. Central cervical lymph nodes are much smaller than lateral lymph nodes and their mean size is less than 1 cm [10], which makes it possible that central cervical lymph nodes might benefit more from the help of SWE. However, the imaging quality and reliability of ultrasound may be influenced by the complicated anatomic structure in central neck such as clavicle, sternum, trachea, carotid artery and so on [11]. To avoid these influences in vivo, lymph nodes were evaluated ex vivo in our study. The aim of this study was to investigate if the SWE could help to differentiate metastatic form non-metastatic level VI lymph nodes in a pre-clinic ex-vivo model in patients with PTC. Because the Hashimoto’s thyroiditis might influence the diagnostic performance [12], lymph nodes from patients without Hashimoto’s thyroiditis were also analyzed separately.

## Methods

### Specimens

From April to May 2017, we collected and scanned 30 consecutive patients’ fresh cervical surgical specimens before rapid frozen sections. All these patients were underwent thyroid surgery and regional cervical lymph nodes resection in our hospital because of confirmed or suspicious malignant thyroid nodules. Among them, 8 patients were excluded because of following reasons: 1. primary thyroid lesions were confirmed to be benign in pathology (5 patients); 2. There were no obvious lymph nodes in ultrasound imaging of the specimens and then pathologic results confirmed that there were no tumor metastases (2 patients); 3. SWE was performed but failed because of the poor quality of SWE (1 patient). Finally, total of 87 level VI lymph nodes from 22 patients, 17 females and 5 males, were enrolled in this study, including 14 level VI lymph nodes from 3 patients with papillary thyroid microcarcinoma (PTMC) and Hashimoto’s thyroiditis, 37 level VI lymph nodes from 7 patients with PTC, and 36 level VI lymph nodes from 12 patients with PTMC. Mean age of these patients was 45.6 ± 13.3 (range 25–70) y. This study was approved by the Independent Ethics Committee of Shanghai Tongren Hospital. All informed consents were obtained from patients.

### Ultrasound examination

**Fig. 1:**
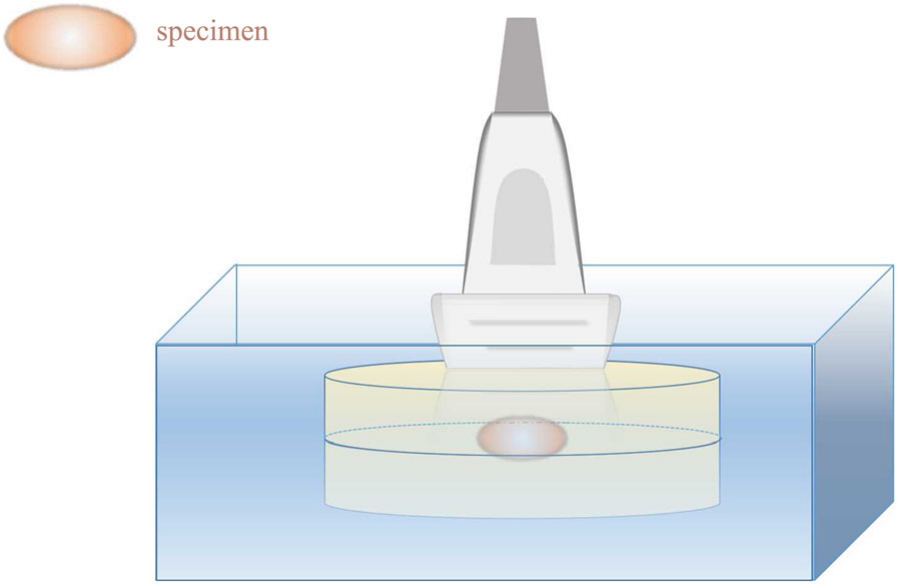
The diagram of ex vivo ultrasound scanning for specimens

All the fresh cervical lymph nodes surgical specimens were scanned by ultrasound before underwent intraoperative frozen sections. Gray-scale ultrasound and SWE were performed using the Aixplore US system (SuperSonic Imaging, Aix-en-Provence, France) with a 15–4-MHz linear-array transducer (SuperLinear SL15–4). Specimens were placed between two gel pads that were putted together in a quadrate glass container (Fig. 1). Ultrasonic coupling agent was used to eliminate the clearance between specimens and gel pads. Gray-scale ultrasound and SWE were performed respectively on entire specimen and corresponding image data were stored as short video files for the sake of subsequent analysis. When performing SWE, probe was placed on the gel pad as lightly as possible to avoid pressure artifact. Imaging quality was considered to be poor when there were less than 80% color signal within the sample frame. Sample frame was selected to ensure that entire specimen was in the central of frame. If the specimen was too big to contain in the sample frame completely, second scan was needed to complete it. All the lymph nodes were evaluated by one radiologist.

### Images analysis

**Fig. 2:**
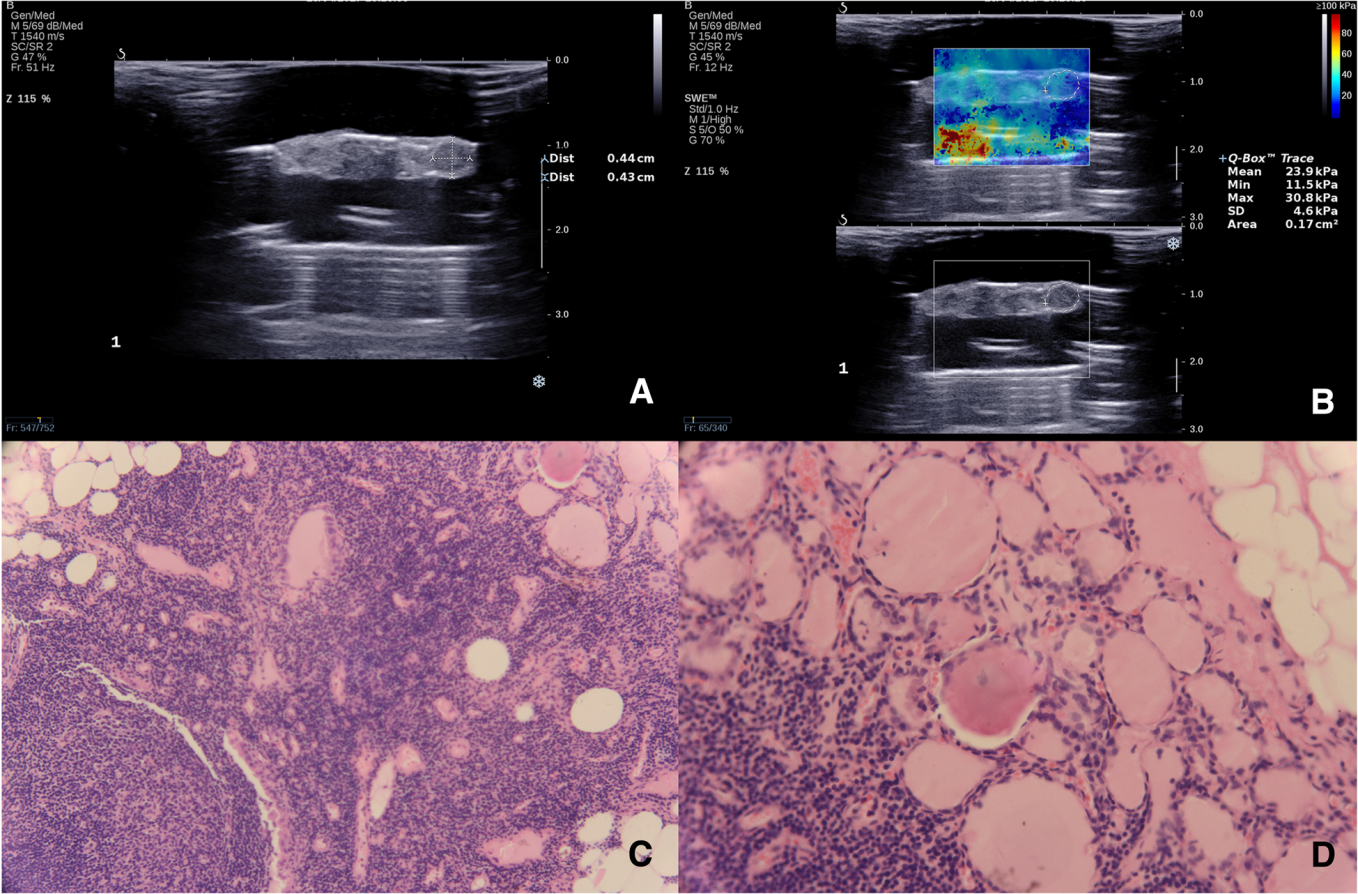
A 61-year-old man with papillary thyroid carcinoma in the right lobe of thyroid. The long diameter and short diameter of one lymph node at ipsilateral level VI were 4.4 mm and 4.3 mm (**a**). S/L ratio was 0.977. E_mean_, E_max_ and E_SD_ were 23.9 kPa, 30.8 kPa and 4.6 kPa respectively (**b**). Gray-ultrasound score and combination score were 2 and 5 respectively. This lymph node was confirmed to be metastatic by pathological diagnosis (**c**, original magnification × 40, and **d**, original magnification × 100)

**Fig. 3:**
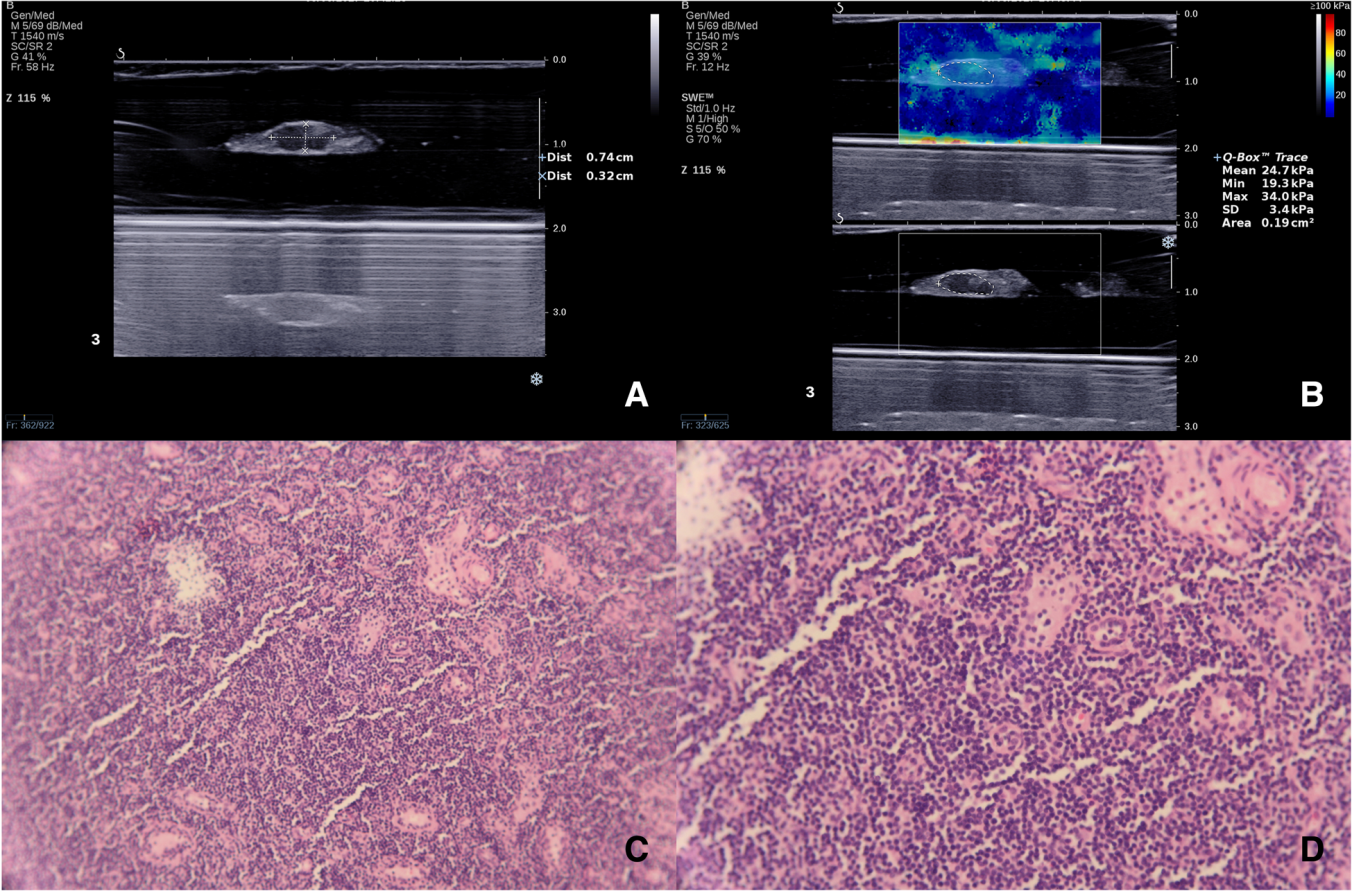
A 54-year-old woman with papillary thyroid microcarcinoma in the left lobe. The long diameter and short diameter of one lymph node at ipsilateral level VI were 4.1 mm and 2.1 mm (**a**). S/L ratio was 0.512. E_mean_, E_max_ and E_SD_ were 18.0 kPa, 23.1 kPa and 2.9 kPa respectively (**b**). Gray-ultrasound score and combination score were 0 and 0 respectively. This lymph node was confirmed to be non-metastatic by pathological diagnosis (**c**, original magnification × 40, and **d**, original magnification × 100)

**Fig. 4:**
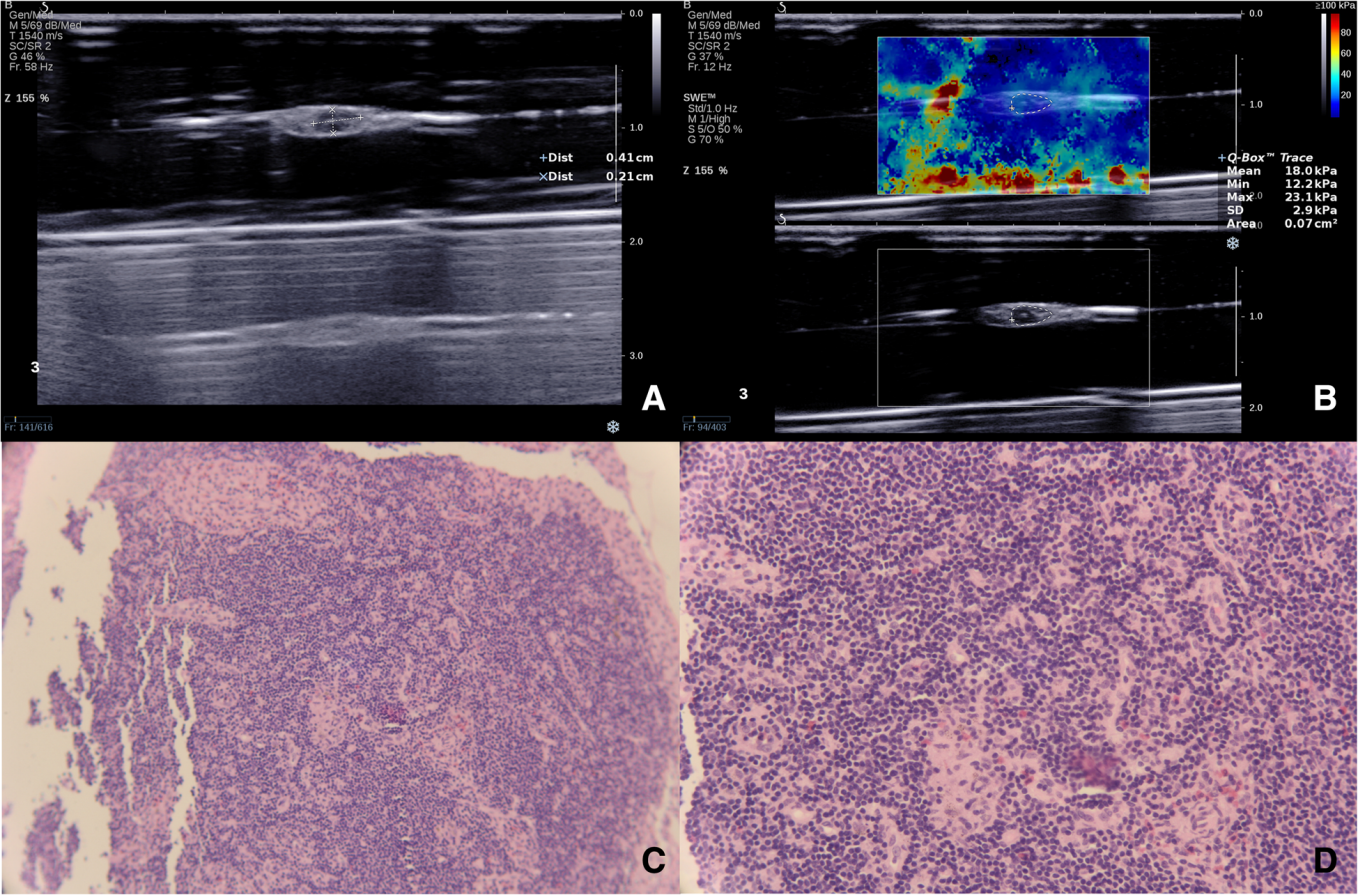
A 40-year-old woman with papillary thyroid microcarcinoma in the right lobe coexisted with Hashimoto’s thyroiditis. The long diameter and short diameter of one lymph node at ipsilateral level VI were 7.4 mm and 3.2 mm (**a**). S/L ratio was 0.432. E_max_ and E_SD_ were 34.0 kPa and 3.4 kPa respectively (**b**). Gray-ultrasound score and combination score were 1 and 2 respectively. This lymph node was confirmed to be non-metastatic by pathological diagnosis (**c**, original magnification × 40, and **d**, original magnification × 100)

US assessment was performed blind of pre-operative staging and pathological staging. The short diameter and long diameter of lymph nodes were measured in their largest section on gray-scale ultrasound and then short-to-long diameter ratio (S/L ratio) was calculated. Elastography indexes, including E_mean_, E_min_, E_max_ and E_SD_, were measured by manually depicting a region of interest (ROI) in which only entire lymph node was contained (Figs. 2, 3 and 4). The four elastography indexes of E_mean_, E_min_, E_max_ and E_SD_ respectively refer to the mean value, minimum value, maximum value and the standard deviation of the Young modulus (represent for elastic modulus) in the ROI.

Intraoperative frozen section results were considered as reference standard to evaluate the diagnostic value of each index (including three gray-scale ultrasound indexes and four SWE indexes). All frozen section results in our study were proved to be consistent with subsequently paraffin section results. Significant indexes and their best cutoff values were determined respectively in all patients and in patients without Hashimoto’s thyroiditis by statistical analysis. Then, a score of 0 or 1 was given for each significant index according to whether they were suspicious for malignancy (score was 1 when it was suspicious for malignancy). Overall scores of gray-scale ultrasound alone and combination of gray-scale ultrasound and SWE were gained respectively to evaluated lymph nodes status.

### Statistical analysis

Statistical analyses were performed with IBM SPSS Statistics version 20.0 software. Quantitative dates were expressed as mean ± standard deviation (Sd) when normal distribution was achieved. Continuous variables were analyzed by t-tests and a value of *p* < 0.05 was considered statistically significant. Receiver operating characteristic (ROC) curves were plotted to assess the diagnostic performance and the best cutoff value of each significant index were determined by calculating Youden index. Comprehensive scores of gray-scale ultrasound, SWE and combination of them were also analyzed by ROC curve. Intraoperative frozen section results were considered as reference standard to calculate the sensitivity, specificity and area under the curve (AUC) of different diagnostic methods.

## Results

### Pathological diagnosis

Pathological diagnosis showed that there were 70 non-metastatic lymph nodes and 17 metastatic lymph nodes in all. Among all lymph nodes, 56 non-metastatic lymph nodes and 17 metastatic lymph nodes were from patients without Hashimoto’s thyroiditis. 22.7% (5/22) patients have level VI lymph nodes metastases, including 4 patients with PTC and 1 patient with PTMC.

### Ultrasound performance of metastatic and non-metastatic lymph nodes

#### Performance of gray-scale ultrasound

Among the three gray-scale ultrasound indexes, short diameter and S/L ratio were significantly different between metastatic and non-metastatic level VI lymph nodes both in all patients and in patients without Hashimoto’s thyroiditis (Table 1). Long diameter was not significant in differentiation between metastatic and non-metastatic level VI lymph nodes.

**Table 1:** Gray-scale ultrasound performance of central cervical lymph nodes

Indexes	Pathological Results	All patients			Patients without Hashimoto’s thyroiditis		
		Number	mean ± Sd	*p*	Number	mean ± Sd	*p*
Long diameter (mm)	(+)	17	4.31 ± 1.20	0.739	17	4.31 ± 1.20	0.621
	(−)	70	4.43 ± 2.02		56	4.07 ± 1.84	
Short diameter (mm)	(+)	17	3.01 ± 1.16	**0.009**	17	3.01 ± 1.16	**0.002**
	(−)	70	2.14 ± 0.81		56	1.93 ± 0.58	
S/L ratio	(+)	17	0.70 ± 0.21	**0.003**	17	0.70 ± 0.21	**0.003**
	(−)	70	0.52 ± 0.13		56	0.52 ± 0.14	

The pathological result of (+) means metastatic and (−) means non-metastatic. Boldface indicates that *p* value is statistically significant

#### Performance of SWE

In all patients, two SWE indexes, E_max_ and E_SD_, were significant in the differentiation of metastatic and non-metastatic level VI lymph nodes (Table 2). In patients without Hashimoto’s thyroiditis, in addition to the E_max_ and E_SD_, the SWE index of E_mean_ was also significant (Table 2).

**Table 2:** SWE performance of central cervical lymph nodes

indexes	Pathological Results	All patients		Patients without Hashimoto’s thyroiditis	
		mean ± Sd	*p*	mean ± Sd	*p*
E_mean_ (kPa)	(+)	22.97 ± 7.75	0.197	22.97 ± 7.75	**0.030**
	(−)	19.87 ± 9.03		17.88 ± 8.44	
E_min_ (kPa)	(+)	13.29 ± 8.41	0.433	13.29 ± 8.41	0.809
	(−)	14.88 ± 7.25		13.80 ± 7.44	
E_max_ (kPa)	(+)	32.72 ± 10.21	**0.016**	32.72 ± 10.21	**< 0.001**
	(−)	25.08 ± 11.80		21.98 ± 9.85	
E_SD_ (kPa)	(+)	4.75 ± 2.83	**0.006**	4.75 ± 2.83	**0.001**
	(−)	2.55 ± 1.81		2.10 ± 1.44	

The pathological result of (+) means metastatic and (−) means non-metastatic. Boldface indicates that p value is statistically significant

### Diagnostic values of significant indexes and different diagnostic methods

The best cutoff values of short diameter, S/L ratio, E_max_ and E_SD_ were 2.55 mm, 0.605, 29.20 kPa and 3.45 kPa respectively for all patients, 2.55 mm, 0.605, 25.65 kPa and 3.45 kPa respectively for patients without Hashimoto’s thyroiditis (Table 3). The best cutoff value of E_mean_ in patients without Hashimoto’s thyroiditis was 21.15 kPa (Table 3). The AUC of E_SD_ was higher than that of other SWE indexes.

**Table 3:** Diagnostic performance of each significant index

	Indexes	Cutoff value	AUC	Sensitivity (%)	Specificity (%)	score	
						0	1
All patients	Short diameter(mm)	2.55	0.729	64.7	78.6	< 2.55	≥2.55
	S/L ratio	0.605	0.772	76.5	80.0	< 0.605	≥0.605
	E_max_ (kPa)	29.20	0.705	70.6	71.4	< 29.2	≥29.2
	E_SD_ (kPa)	3.45	0.767	70.6	84.3	< 3.45	≥3.45
Patients without Hashimoto’s thyroiditis	Short diameter(mm)	2.55	0.783	64.7	91.0	< 2.55	≥2.55
	S/L ratio	0.605	0.769	76.5	76.8	< 0.605	≥0.605
	E_mean_ (kPa)	21.15	0.690	76.5	73.2	< 21.15	≥21.15
	E_max_ (kPa)	25.65	0.779	76.5	76.8	< 25.65	≥25.65
	E_SD_ (kPa)	3.45	0.822	70.6	91.1	< 3.45	≥3.45

The AUC of combination method was higher than the AUC of gray-scale ultrasound both in all patients (0.887 vs. 0.841) and in patients without Hashimoto’s thyroiditis (0.925 vs. 0.866). The AUC of each diagnostic method in patients without Hashimoto’s thyroiditis was higher than that in all patients (Table 4).

**Table 4:** Diagnostic performance of gray-scale ultrasound and combination method

	Methods	Best cutoff value	Sensitivity (%)	Specificity (%)	AUC
All patients	Gray-scale ultrasound	1.5	52.9	98.6	0.841
	Combination method	1.5	82.4	77.1	0.887
Patients without Hashimoto’s thyroiditis	Gray-scale ultrasound	0.5	88.2	67.9	0.866
	Combination method	1.5	94.1	76.8	0.925

## Discussion

PTC tends to involve cervical lymph nodes with the incidence of metastases ranges from 20 to 90% [13]. In our study, the incidence of level VI lymph nodes metastases was 22.7% in all. The low incidence of metastases might result from more PTMC enrolled in our study. PTMC, which is defined by the World Health Organization (WHO) as a PTC 1.0 cm or smaller in its maximal diameter, has lower incidence of metastases than tumors that size was more than 1.0 cm [13, 14]. Although ultrasound did not detect all the lymph nodes that were dissected in present study, the number of non-metastatic lymph nodes was 4 times that of metastatic lymph nodes (70 vs. 17) in present study, which was similar to previous study [15].

High-resolution ultrasound has been widely applied to the preoperative evaluation of cervical lymph nodes and it might be the best methodology for determining subsequent surgical treatment of cervical lymph nodes in patients with PTC [16]. Nodal size has always been used as an ultrasound indicator to differentiate the benign and malignant cervical lymph nodes [17]. In our study, the results demonstrated that metastatic level VI lymph nodes had longer short diameters than non-metastatic lymph nodes both in all patients (*p* = 0.009) and in patients without Hashimoto’s thyroiditis (*p* = 0.002). Level VI lymph nodes were much smaller than lateral lymph nodes [10]. Therefore, the cutoff value of size in level VI might be different from others. In previous studies, 5 mm was used as the best cutoff value of level VI lymph nodes in vivo and had low sensitivity [14]. The best cutoff value of short diameter in our study was 2.55 mm that was much smaller than previous in vivo studies, which might result from much more small lymph nodes detected in condition of ex vivo. The sensitivity of short diameter was moderate. S/L ratio was the other significant gray-scale index in our study and the best cutoff value was 0.605. Shape of lymph nodes tended to be round when the S/L ratio became larger. And round shape had been proved to be an indicator of malignant cervical lymph nodes [18, 19]. Previous studies also used other indicators such as echogenicity, microcalcification, nodal vascularity and so on to evaluate cervical lymph nodes [15, 20]. However, the lymph nodes detected in our study were too small to accurately observe other features. Besides, the vascularity could not be detected due to ex vivo condition. Combining two significant indexes of short diameter and S/L ratio, gray-scale ultrasound had low sensitivity (52.9%) and high specificity (98.6%) in all patients, which was similar to previous studies [21, 22]. In patients without Hashimoto’s thyroiditis, gray-scale ultrasound had high sensitivity (88.2%) and moderate specificity (67.9%).

SWE can quantitatively measure tissue elasticity and has been confirmed to be a good complementary tool with high diagnostic accuracy for head and neck lymph nodes, especially for sub-centimeter cervical lymph nodes [8, 9]. In our study, metastatic level VI had higher E_max_ (*p* = 0.016) and E_SD_ (*p* = 0.006) in all patients. In patients without Hashimoto’s thyroiditis, metastatic level VI lymph nodes had higher E_mean_ (0.030), E_max_ (*p* < 0.001) and E_SD_ (*p* = 0.001) (Figs. 2 and 3). Previous studies used E_max_ to evaluate cervical lymph nodes considering that E_max_ might reflect the heterogeneous histology better than E_mean_ and the other indexes [9, 23]. Higher E_mean_ had also been used as an indicator of malignant cervical lymph nodes while it had low sensitivity [24]. However, E_SD_ had never been used as an indicator. In our study, we found that E_SD_ had the highest AUC among all SWE indexes both in all patients (0.767) and patients without Hashimoto’s thyroiditis (0.822). The present study confirmed that metastatic lymph nodes were stiffer than non-metastatic and that E_SD_ might be the best SWE index for the evaluation of level VI lymph nodes. Higher E_SD_ in metastatic lymph nodes might be caused by more heterogeneous components contained in them such as microcalcification, necrosis and cystic changes [19]. The sensitivity of individual index alone was not high. Combining all significant SWE indexes with significant gray-scale ultrasound indexes, combination method had higher AUC than gray-scale in all patients (0.887 vs. 0.841). In patients without Hashimoto’s thyroiditis, combination method improved the diagnostic sensitivity (94.1% vs. 88.2%), specificity (76.8% vs. 67.9%) and AUC (0.925 vs. 0.866) when compared with gray-scale ultrasound. In general, our study demonstrated that SWE might provide additional information to help the evaluation of level VI lymph nodes in patients with PTC, which was similar to the results of previous studies [25].

Hashimoto’s thyroiditis, which is also called chronic autoimmune thyroiditis, may be a negative predictive factor for level VI lymph nodes metastases in patients with PTC [26, 27]. In our study, 14 level VI lymph nodes in 3 patients with coexistence of PTC and Hashimoto’s thyroiditis were all non-metastatic. Hashimoto’s thyroiditis may influences the features of cervical lymph nodes [28] and previous study had demonstrated that the diagnostic performance of ultrasound for level VI lymph nodes metastases was lower in PTC with Hashimoto’s thyroiditis than that in PTC without Hashimoto’s thyroiditis [12]. In present study, we analyzed all indexes and different diagnostic methods respectively in all patients and patients without Hashimoto’s thyroiditis and found that the AUCs of short diameter (0.783 vs. 0.729), E_max_ (0.779 vs. 0.705) and E_SD_ (0.822 vs. 0.767) were all higher in patients without Hashimoto’s thyroiditis. The AUCs of gray-scale ultrasound (0.866 vs. 0.841) and combination method (0.925 vs. 0.887) were all higher in patients without Hashimoto’s thyroiditis than that in all patients. Our study indicated that the Hashimoto’s thyroiditis might influence the diagnostic performance of both gray-scale ultrasound and SWE for level VI lymph nodes in patients with PTC (Fig. 4) and that diagnostic performance of ultrasound and SWE might higher in patients without Hashimoto’s thyroiditis.

This study had several limitations. First, the sample was small because the acquisition of surgical specimens before rapid frozen section was difficult. Present study showed the potential value of SWE in the assessment of level VI lymph nodes. However, large sample studies are needed to confirm the results further. Second, our study was performed in the condition of ex vivo which could not completely represent the condition in vivo. However, it could unify the surrounding environment and made the ultrasound imaging and results more accurate and reliable. Clinical studies in vivo are still essential and in progress. Third, measurements were performed by one radiologist, and inter-observer and intra-observer variations were not estimated in our study. However, SWE had been proved to have high reproducibility [9]. Therefore, it may not impact the final results. What’s more, due to the limitation of ex-vivo condition, only three gray-scale ultrasound parameters were included, which might miss some important characteristics. Methods should be optimized in subsequent in-vivo researches. Besides, our study showed that the diagnostic performance of gray-scale ultrasound and SWE for level VI lymph nodes metastases in PTC tended to be better in patients without Hashimoto’s thyroiditis. Studies should be performed in the future focusing on the influence of Hashimoto’s thyroiditis to the ultrasound features of metastatic cervical lymph nodes in PTC.

## Conclusion

Our study demonstrated that shear wave elastography can provide additional information to help ultrasound evaluation of level VI lymph nodes in papillary thyroid carcinoma, especially in papillary thyroid carcinoma without Hashimoto’s thyroiditis.

## Abbreviations

ATA: American Thyroid Association
AUC: Area Under the Curve
PTC: Papillary Thyroid Carcinoma
PTMC: Papillary Thyroid Microcarcinoma
ROC curve: Receiver Operating Characteristic
S/L ratio: Short-to-Long diameter ratio
SWE: Shear Wave Elastography
